# Association between Subjective and Objective Assessment of Enucleation Outcome Depending on the Presence of an Orbital Implant in Patients with Uveal Melanoma

**DOI:** 10.3390/jcm11082141

**Published:** 2022-04-12

**Authors:** Weronika Pociej-Marciak, Bożena Romanowska-Dixon, Katarzyna Żuber-Łaskawiec, Mojca Globočnik Petrovič, Izabella Karska-Basta

**Affiliations:** 1 Division of Ophthalmology and Ocular Oncology, Department of Ophthalmology, Faculty of Medicine, Jagiellonian University Medical College, 31-501 Kraków, Poland; romanowskadixonbozena1@gmail.com (B.R.-D.); kzuberlaskawiec@gmail.com (K.Ż.-Ł.); izabasta@gmail.com (I.K.-B.); 2 Department of Ophthalmology and Ocular Oncology, University Hospital, 31-501 Kraków, Poland; 3 Eye Hospital University Medical Centre Ljubljana, Faculty of Medicine, University of Ljubljana, 1000 Ljubljana, Slovenia; mgpetrovic@yahoo.com

**Keywords:** cosmetic outcome, prosthetic eye, objective assessment, orbital implant, subjective assessment, uveal melanoma

## Abstract

We aimed to assess the cosmetic outcome of patients who underwent enucleation for uveal melanoma. The subjective assessment was based on a questionnaire, including four questions on postoperative cosmetic outcome. As part of the objective assessment, the following features were evaluated using a four-point scale: the symmetry of the upper eyelid sulcus, color matching between the prosthetic and healthy eye, prosthetic eye motility, and eyelid position. We enrolled 90 patients after enucleation (58 with and 32 without an orbital implant). The overall subjective assessment scores were 3.5/4 and 3.3/4 points in patients with and without an implant, respectively. The overall objective assessment scores were 3.3/4 and 2.3/4 in patients with and without an implant, respectively (*p* < 0.001). The cosmetic outcome was rated significantly higher by patients than by investigators (*p* < 0.05). There was no significant association between the overall subjective and objective assessment of the cosmetic outcome in any of the groups. Cosmetic outcome after enucleation for uveal melanoma was highly rated by patients. It was rated higher by patients than by investigators. The presence of an orbital implant was associated with higher objective assessment scores in terms of the symmetry of the upper lid sulcus, prosthetic eye motility, and eyelid position.

## 1. Introduction

Uveal melanoma is the most common primary intra-ocular tumor in adults [1,2,3]. In recent decades, there has been considerable progress in eye-sparing treatment modalities for uveal melanoma (such as proton radiotherapy, brachytherapy, transpupillary thermotherapy, and CyberKnife radiotherapy). However, the enucleation of the eye is still required in some cases [4]. Enucleation is necessary in the presence of the following tumor features: a large tumor size (thickness above 10 mm; diameter above 18 mm), the presence of extra-ocular extension, the tumor invasion of the optic disc or ring melanoma involving more than half of the circumference of the eye, radiotherapy complications (secondary glaucoma, ocular pain, and phthisis bulbi), recurrence after brachytherapy, and poor response to brachytherapy [5,6].

One of the complications of enucleation is so-called postenucleation socket syndrome (PESS), which most often occurs after enucleation carried out without using an orbital implant [7]. The symptoms of PESS include enophthalmos, deep upper eyelid sulcus, eyelid dysfunction (lagophthalmos, ptosis, and retraction), loss of the inferior fornix, and laxity of the lower eyelid [7,8,9]. To prevent PESS, orbital implants have been developed. Orbital implantation in an eviscerated globe was first described by Mules in 1885 [10,11,12,13,14]. In 1886, Frost placed a similar implant in a patient after enucleation [9,13,14,15]. Since then, various orbital implants have been used to improve the cosmetic outcome of enucleation and increase the motility of the prosthesis [10,16].

After enucleation, both with and without fitting an orbital implant, it is necessary to fabricate a customized prosthetic eye. Prosthetic eyes aim to improve the cosmetic effect of the surgery and prevent changes in the orbital anatomy [12]. Currently, the most widely available prosthetic eyes are made of cryolite glass (which becomes translucent at high temperatures) and acryl (polymethyl methacrylate). The use of silicone ocular prostheses remains controversial [14].

So far, studies in patients undergoing enucleation for uveal melanoma have mainly focused on the assessment of patient survival and the histopathological characteristics of melanomas [17,18]. Only a few studies have evaluated quality of life in this population of patients [19,20]. Moreover, data on the cosmetic outcome of enucleation for uveal melanoma are lacking. Today, with the increasing focus on appearance, it is necessary to ensure that all patients can wear a realistic, natural, and comfortable prosthetic eye after enucleation. The aim of this study was to assess the subjective and objective cosmetic outcomes of enucleation in patients with uveal melanoma.

## 2. Materials and Methods

This study included patients after enucleation for uveal melanoma who presented for a follow-up visit to the Department of Ophthalmology, Division of Ophthalmology and Ocular Oncology, Jagiellonian University Medical College, Krakow, between May 2015 and January 2017. The inclusion criteria were as follows: patients aged above 18 years, with previous enucleation for uveal melanoma, and with written informed consent to participate in the study. Patients with concomitant cancer (remission period < 12 months) and those unable to respond to the questionnaire or have photographs taken due to a psychosomatic condition were excluded. The research group included a psychologist. The study was approved by the Jagiellonian University Bioethics Committee (No. 122.6120.5.2015). 

### 2.1. Subjective Assessment of Cosmetic Outcome

The subjective assessment of the cosmetic outcome was based on a short questionnaire, including the following four questions rated on a four-point scale:

- “Are you satisfied with the cosmetic effect of the ocular prosthesis?” (Subjective satisfaction with the cosmetic effect of the ocular prosthesis.)
- “Does the color of the prosthetic eye match the color of the healthy eye?” (Subjective assessment of the color matching between the prosthetic and healthy eye.)
- “Is the motility of the prosthetic eye similar to that of the healthy eye?” (Subjective assessment of the prosthesis motility.)
- “Does the prosthetic eye happen to slip out of place during daily activities?” (Subjective assessment of the fit of the prosthetic eye.)

The first three questions were rated according to the following scale: 1—not at all; 2—partly; 3—significantly; and 4—highly. For the fourth question, the following scale was used: 1—very often; 2—quite often; 3—sometimes; and 4—not at all. To obtain the overall subjective assessment score, the points for each question were summarized and then divided by four. 

### 2.2. Objective Assessment of Cosmetic Outcome

The objective assessment of the cosmetic outcome was based on the photographs of healthy and prosthetic eyes in five directions of gaze (straight ahead, to the right, to the left, up, and down). The photographs were taken with the patient’s head immobilized (Nikon 2700, 18-105 VR Kit, Tokyo, Japan) and the camera attached to a tripod. A scale ruler was placed near the patient’s eye and photographed, which allowed us to determine the scale for converting pixels into millimeters. The following features were assessed based on the obtained photographs: the symmetry of the upper eyelid sulci, color matching between the painted iris of the prosthetic eye and the iris of the healthy eye, and the motility of the prosthetic and healthy eye, as well as eyelid position.

Symmetry of the upper eyelid sulci was assessed on the basis of the straight-ahead direction using a four-point scale: 1—asymmetrical; 2—deepening of more than half of the sulcus; 3—deepening of less than half of the sulcus; and 4—symmetrical.

Color matching between the prosthetic and healthy eye was assessed on the basis of the straight-ahead direction using a four-point scale, where 1—similarity; 2—slight similarity; 3—significant similarity; and 4—high similarity. The color was assessed by three investigators using the same light conditions and monitor display settings. Two extreme scores were rejected, while the middle score was considered as denoting an adequate assessment of the color matching. In the case of two identical scores, they were considered a proper assessment of the color matching.

The motility of the prosthetic and healthy eye was measured in the vertical and horizontal directions using the Image J software (National Institutes of Health, Bethesda, Maryland, and the Laboratory for Optical and Computational Instrumentation, University of Wisconsin, Wisconsin, United States). First, we measured iris deviation in the four directions of gaze: temporal, nasal, superior, and inferior. The results were expressed in millimeters. Due to the naturally higher eye motility in the horizontal than in the vertical direction (about 10 mm vs. 7 mm), the results were categorized as follows: for the horizontal direction (temporal and nasal): 0–0.75 mm—no (1); 0.76–1.5 mm—slight (2), 1.51–2.25 mm—significant (3), and ≥2.26 mm—high (4); for the vertical direction (up and down): 0–0.5 mm—no (1); 0.51–1.25 mm—slight (2); 1.26–2.00 mm—significant (3); and ≥2.01 mm—high (4).

**Figure 1:**
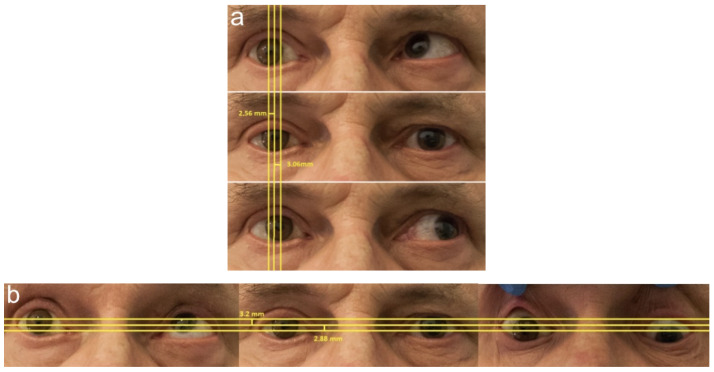
Measurement of eye motility in millimeters: (**a**) the horizontal direction (temporal and nasal); (**b**) the vertical direction (up and down) using a previously described method by Guthoff et al. [21]. Source: Division of Ophthalmology and Ocular Oncology, Jagiellonian University Medical College; own material.

The measurement of eye motility in millimeters is presented in Figure 1 (using a previously described method by Guthoff et al. [21]).

Eyelid position was assessed from the straight-ahead direction based on the presence of ptosis/upper eyelid retraction or lower eyelid ectropion/laxity using the four-point scale: 1—abnormal; 2—partially abnormal; 3—mostly normal; and 4—normal. The eyelids were assessed by three investigators using the same lighting conditions and monitor display settings. Two extreme scores were rejected, while the middle score was considered as the proper assessment of the eyelid position. In the case of two identical scores, they were considered as denoting a proper assessment of the eyelid position. The overall objective assessment score was obtained by summarizing the scores for all four questions (sulcus symmetry, color matching, prosthesis motility, and eyelid position) and dividing the sum by four. 

**Figure 2:**
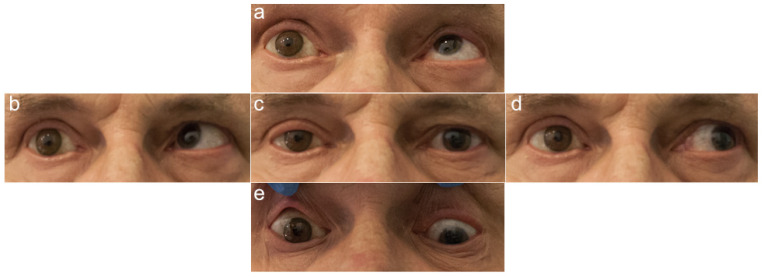
Photographs of a patient with an orbital implant and a glass prosthetic eye on the right; five cardinal directions of gaze: (**a**) up, (**b**) to the right, (**c**) straight ahead, (**d**) to the left, and (**e**) down. Source: Division of Ophthalmology and Ocular Oncology, Jagiellonian University Medical College; own material.

**Figure 3:**
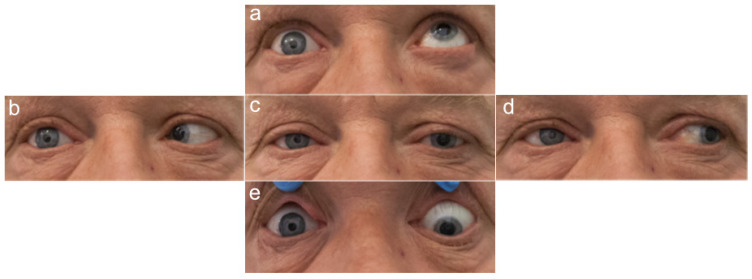
Photographs of a patient with an orbital implant and an acrylic prosthetic eye on the right. Five cardinal directions of gaze: (**a**) up, (**b**) to the right, (**c**) straight ahead, (**d**) to the left, and (**e**) down. Source: Division of Ophthalmology and Ocular Oncology, Jagiellonian University Medical College; own material.

**Figure 4:**
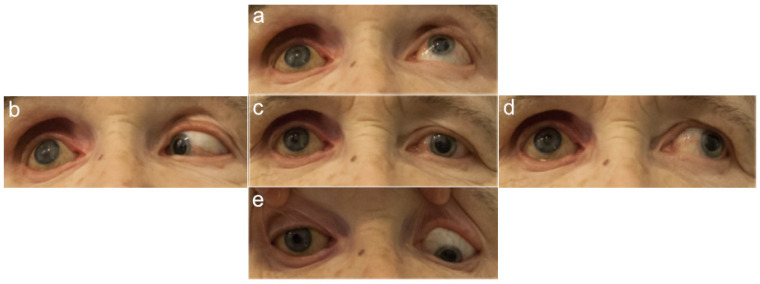
Photographs of a patient without an orbital implant, with a glass prosthetic eye on the right. Five cardinal directions of gaze: (**a**) up, (**b**) to the right, (**c**) straight ahead, (**d**) to the left, and (**e**) down. Source: Division of Ophthalmology and Ocular Oncology, Jagiellonian University Medical College; own material.

**Figure 5:**
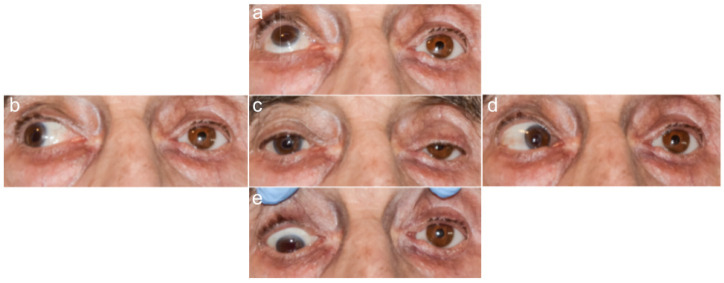
Photographs of a patient without an orbital implant, with an acrylic prosthetic eye on the left; five cardinal directions of gaze: (**a**) up, (**b**) to the right, (**c**) straight ahead, (**d**) to the left, and (**e**) down. Source: Division of Ophthalmology and Ocular Oncology, Jagiellonian University Medical College; own material.

The investigators who assessed the photographs and performed the measurements were blinded to patient data, such as the presence of an orbital implant and the type of prosthetic eye. The photographs of the healthy and prosthetic eyes in the five cardinal directions of gaze are presented in Figure 2, Figure 3, Figure 4 and Figure 5.

### 2.3. Statistical Analysis

Qualitative variables were presented as absolute numbers and percentages. Quantitative variables were presented as a mean with standard deviation, and, in the case of a non-normal distribution, as a median of the first and third quartiles. 

Contingency tables were used to present associations between the qualitative variables. Significant associations between variables were assessed using the Pearson chi-squared test if the expected numbers were higher than five in at least 80% of the cells. Otherwise, the Fisher test was used for 2 × 2 tables and the Fisher–Freeman–Halton test for the remaining tables. For comparisons between unrelated groups, the Mann–Whitney test was used for interval dependent variables and the Kolmogorov–Smirnoff test was used for ordinal dependent variables. For comparisons between related groups, the Wilcoxon test was used for both interval and ordinal-dependent variables. 

The Spearman rho correlation coefficient was used to assess the strength of correlations between interval variables. The loess regression curve was applied to graphically present the correlations between variables measured on at least the ordinal scale. 

The linear regression model analysis was used to identify factors affecting the overall objective assessment of the cosmetic outcome and the quality of life. A *p* value of less than 0.05 was considered significant. The statistical analysis was performed using IBM SPSS Statistics 24 for Windows.

## 3. Results

The study included 90 patients: 44 women and 46 men. The mean age of the study population was 60.9 years (SD, 13.5 years). There were 32 patients without an orbital implant and 58 patients with an orbital implant (Guthoff orbital implants, size 20 mm, in all patients). An acrylic prosthetic eye was used by 29 patients and a glass prosthetic eye by 61 patients. The mean time since enucleation was 62.5 months (SD, 55.2 months), and the mean time since the fitting of the prosthetic eye was 3 years (SD, 3.0 years). The demographic and clinical characteristics of patients with and without an orbital implant are presented in Table 1.

### 3.1. Subjective Assessment of Cosmetic Outcome

In the subjective assessment, prosthesis motility and fitting were rated higher by patients with an orbital implant than by those without (*p* < 0.05). The results of the subjective assessment in both groups are presented in Table 2.

### 3.2. Objective Assessment of Cosmetic Outcome

Asymmetry of the upper eyelid sulcus was greater in patients without an orbital implant than in those with an implant (*p* < 0.001). The results of the analysis in both groups are presented in Table 3. 

Of the study population, 76 patients (84.4%) had blue eyes and 14 patients (15.6%) had brown eyes. The assessment of color matching between the prosthetic and healthy eye in patients with and without an orbital implant is presented in Table 4.

In the objective assessment, prosthesis motility in the vertical and horizontal directions was rated higher by investigators in all directions of gaze in patients with an orbital implant than in those without an implant (Table 5).

The assessment of eyelid position is presented in Table 6. There were significant differences in eyelid position between patients with and without an orbital implant. Patients without an implant more often showed abnormalities in eyelid position (Table 6).

### 3.3. Subjective and Objective Assessment of Cosmetic Outcome

The overall score in the objective assessment of cosmetic outcome was higher in patients with an orbital implant than in those without an implant (*p* < 0.001). On the other hand, there were no differences in the overall subjective assessment score between groups (*p* > 0.05). The overall subjective assessment score was higher than the overall objective assessment score both in patients with and without an orbital implant (*p* < 0.05) (Table 7).

**Figure 6:**
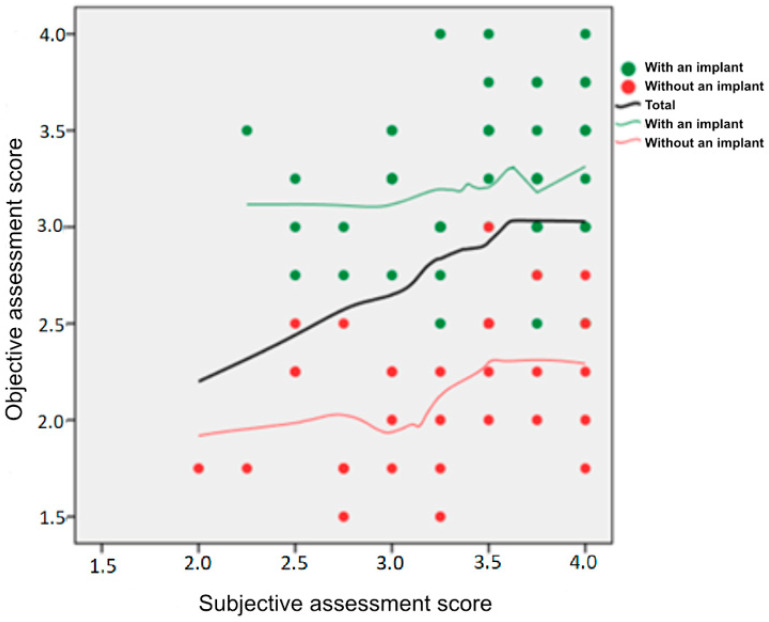
A scatterplot of a correlation between the overall objective and subjective assessment scores in patients with and without an orbital implant. The loess curves represent the mean objective score for a given level of the subjective score.

The linear regression model analysis showed that the presence of an orbital implant was significantly associated with a higher overall objective assessment score, irrespective of the subjective assessment score (Figure 6).

## 4. Discussion

### 4.1. Subjective Assessment of Cosmetic Outcome

A subjective assessment of cosmetic outcome is made by the patient. After enucleation, some patients are not satisfied with the aesthetic effect even though the surgical outcome is good [22]. This dissatisfaction may result from unrealistic expectations or inaccurate information about the possible surgical outcome [22]. 

In this study, the cosmetic outcome of enucleation was highly rated by patients. The questions regarding the level of satisfaction, color matching, and the fit of the prosthetic eye scored a median of four points (out of a possible four), both in the group with and without an orbital implant. Only prosthesis motility scored a median of three points in patients with an orbital implant and a median of two points in those without an implant. The overall median score in the subjective assessment of cosmetic outcome was similar between patients with and without an orbital implant (3.5 and 3.3 points, respectively). 

In a PhD thesis, Rasmussen assessed the level of satisfaction among patients after enucleation for various causes, including neoplasm (37% of cases). Satisfaction with the cosmetic outcome was reported by 86% of patients, of whom 33% were very satisfied and 53% were satisfied. Only 14% were dissatisfied or very dissatisfied [12]. Song et al. [22] evaluated satisfaction with ocular prosthesis in a Korean population of patients after enucleation or evisceration. The overall rate of satisfaction was 71.8%. The high overall rating of the cosmetic outcome in our study can probably be explained by the fact that patients were aware that the main objective of enucleation was the treatment of cancer. In contrast, in the study by Song et al. [22], only 3 of the 78 patients underwent eye removal due to cancer. 

### 4.2. Objective Assessment of Cosmetic Outcome

Most of the available studies evaluating the cosmetic outcome of enucleation focused on the motility of an orbital implant or a prosthetic eye [21,23,24,25,26,27,28]. Some investigators also assessed other postoperative characteristics of the orbit related to the cosmetic outcome [12,16,29,30]. Rasmussen recorded the presence of exophthalmos and enophthalmos in the amputated eye, the depth of the superior sulcus, horizontal and vertical displacement of the enucleated eye, prosthesis motility, visual acuity, local infection, exposure of the implant, as well as the depth of the superior and inferior fornix [12]. On the other hand, Sassmannshausen et al. [29] assessed the postoperative volume deficit in the orbit, enophthalmos of the prosthesis, the depth of the upper lid sulcus, levator function, and motility of the prosthetic eye in patients undergoing enucleation for uveal melanoma. Mourits et al. [16] studied patients after enucleation for retinoblastoma and assessed the position of the upper and lower eyelid, volume deficiency, prosthetic eye movement, and overall cosmetic appearance.

In our study, we assessed the symmetry of the upper eyelid sulci, color matching between the prosthetic and healthy eye, prosthesis motility, and eyelid position. In patients with an orbital implant, upper eyelid sulci were more symmetrical than in those without an implant. Our results are in line with the study by Sassmannshausen et al. [29], who reported that patients with no implant developed a significantly more pronounced upper lid sulcus than those with an implant. This is related to a greater severity of PESS in patients without an implant versus those with an implant.

In our study, patients with an orbital implant rated the color matching higher than those without an implant. It is possible that the presence of an implant translates to a better assessment of the prosthesis color, because the enophthalmos of the prosthesis is less pronounced in patients with an implant than in those without. Moreover, the assessment of color matching may be affected by the time since implantation of the prosthesis, which was significantly longer in patients without an implant [14]. 

Various methods are used to assess the motility of an orbital implant or a prosthetic eye [12,21,23,24,25,26,27,28,29]. In our study, we used the method previously described by Guthoff et al. [21]. The motility in the four cardinal directions of gaze in patients with an orbital implant was rated significantly better than in patients without an orbital implant, which is in line with other studies [27,29]. None of the patients used a pegged prosthesis, which might have affected the results [21,26]. 

Abnormal eyelid position often indicates the poor fitting of the prosthesis. The orbital fat tissue volume changes over time [8,31]. Moreover, the prosthetic material gradually wears off. Therefore, regular exchange of the prosthesis is recommended [12,14]. When assessing the eyelid position, our results showed that ptosis, upper eyelid retraction, and lower eyelid ectropion/laxity were more common in patients without an orbital implant than in those with an implant. This is in line with the study by Sassmannshausen et al. [29], who showed that lower eyelid laxity and worsening of the levator function resulting in ptosis were more common in patients without an orbital implant than in those with an implant. 

In our study, the overall score in the objective assessment of cosmetic outcome was higher in patients with an orbital implant than in those without an implant. This is in line with the results of other authors [27,29,32].

### 4.3. Subjective vs. Objective Assessment of Cosmetic Outcome

Our study revealed that the cosmetic outcome of enucleation is rated higher in the subjective assessment by patients than in the overall objective assessment by operators, irrespective of the presence of an orbital implant. Song et al. [22] also showed a higher level of satisfaction with the cosmetic outcome among patients than among surgeons. 

In our study, we did not observe any association between the overall subjective and the overall objective assessment of the cosmetic outcome, irrespective of the presence of an orbital implant. However, the presence of an implant significantly improved the objective assessment of cosmetic outcome. Our results are in line with the study of Mourtis et al. [16], who reported that the use of an orbital implant is associated with a better outcome in terms of volume deficit, upper eyelid sulcus depth, lower eyelid position, and prosthesis motility.

This study has several limitations. First, the objective assessment of the cosmetic outcome did not include the evaluation of volume deficit or inferior fornix depth. However, the aim of this study was to perform an objective assessment based on photographs only. This is in line with a study by Lucci et al. [28], who also assessed the cosmetic outcome of enucleation solely on the basis of photographs. Second, the questions relating to subjective assessment only partly corresponded to the parameters assessed by investigators. Finally, the time from enucleation to the assessment of the cosmetic outcome differed between individual patients. However, in our opinion, these limitations do not hinder the subjective and objective assessment of the cosmetic outcome or the analysis of the results.

## 5. Conclusions

Patients after enucleation for uveal melanoma highly rated their postoperative cosmetic outcome. Moreover, the cosmetic outcome was rated higher by patients than by investigators. The presence of an orbital implant was associated with better cosmetic outcome—it improved the symmetry of the upper eyelid sulcus, prosthesis motility, and eyelid position. We did not observe any relationship between the overall subjective assessment and the overall objective assessment of the cosmetic outcome, irrespective of the presence of an orbital implant. However, the presence of the implant significantly improved the objective assessment of the cosmetic outcome. The subjective and objective assessment of the cosmetic outcome is an important issue and should be part of routine clinical practice. Each patient should be provided with multidisciplinary care after enucleation, including regular replacement and individual adjustment of the prosthesis. 

## Figures and Tables

**Table 1:** Characteristics of patients after enucleation with and without an orbital implant.

Parameter	With an Implant	Without an Implant	*p*
Sex, *n* (%)			0.876
Female	28 (48.3)	16 (50)	
Male	30 (51.7)	16 (50)	
Age, mean (SD)	58.9 (13.8)	64.6 (12.2)	0.165
Type of prosthetic eye, *n* (%)			0.883
Glass	39 (67.2)	22 (68.8)	
Acrylic	19 (32.8)	10 (31.3)	
Time since enucleation, mo, median (Q1–Q3)	29.5 (14–49)	108 (83–138.5)	<0.001
Time since the fitting of the prosthetic eye, y, median (Q1–Q3)	1 (1–3)	5 (1–7)	<0.001

A *p* value of less than 0.05 was considered significant.

**Table 2:** Comparison of subjective assessment score between patients with and without an orbital implant.

Subjective Assessment	With an Implant, Median (Q1–Q3)	Without an Implant, Median (Q1–Q3)	*p*
Subjective satisfaction with the cosmetic effect of the ocular prosthesis	4 (3–4)	4 (3–4)	0.588
Subjective assessment of the color matching	4 (3–4)	4 (3–4)	0.635
Subjective assessment of prosthesis motility	3 (2–4)	2 (2–3)	0.002
Subjective assessment of prosthesis fitting	4 (4–4)	4 (3–4)	0.035

A *p* value of less than 0.05 was considered significant.

**Table 3:** Assessment of superior sulcus symmetry in patients with and without an orbital implant.

Superior Sulcus Symmetry	With an Implant, *n* (%)	Without an Implant, *n* (%)	*p*
1—asymmetrical	0	10 (31.3)	<0.001
2—deepening of more than half of the sulcus	3 (5.2)	19 (59.4)	
3—deepening of less than half of the sulcus	28 (48.3)	3 (9.4)	
4—symmetrical	27 (46.6)	0	

A *p* value of less than 0.05 was considered significant.

**Table 4:** Assessment of color matching between prosthetic and healthy eye in patients with and without an orbital implant.

Color Matching	With an Implant, *n* (%)	Without an Implant, *n* (%)	*p*
1—no similarity	3 (4.9)	9 (31.0)	<0.001
2—slight similarity	8 (13.1)	7 (24.1)	
3—significant similarity	35 (57.4)	12 (41.4)	
4—high similarity	15 (24.6)	1 (3.4)	

A *p* value of less than 0.05 was considered significant.

**Table 5:** Assessment of prosthesis motility in patients with and without an orbital implant.

Objective Motility (mm)	With an Implant, Median (Q1–Q3)	Without an Implant, Median (Q1–Q3)	*p*
Temporal	2.05 (1.49–2.40)	0.50 (0.30–0.70)	<0.001
Nasal	2.05 (1.62–2.46)	0.63 (0.35–0.83)	
Superior	1.63 (1.49–1.98)	0.28 (0.00–0.63)	
Inferior	2.01 (1.60–2.35)	0.52 (0.20–0.63)	

A *p* value of less than 0.05 was considered significant.

**Table 6:** Assessment of eyelid position in patients with and without an orbital implant.

Eyelid Position	With an Implant, *n* (%)	Without an Implant, *n* (%)	*p*
1—abnormal	0	1 (3.1)	<0.001
2—partially abnormal	8 (13.8)	11 (34.4)	
3—mostly normal	22 (37.9)	19 (59.4)	
4—normal	28 (48.3)	1 (3.1)	

A *p* value of less than 0.05 was considered significant.

**Table 7:** Subjective and objective assessment of cosmetic outcome: overall scores in patients with and without an orbital implant.

Overall Assessment	With an Implant, Median (Q1–Q3)	Without an Implant, Median (Q1–Q3)	*p*
Overall subjective assessment	3.5 (3.3–3.8)	3.3 (2.8–3.6)	0.294
Overall objective assessment	3.3 (3.0–3.5)	2.3 (1.8–2.5)	<0.001
*p*	<0.001	0.001	---

A *p* value of less than 0.05 was considered significant.

## Data Availability

Not applicable.
